# Effects of different agronomic practices on the selective soil properties and nitrogen leaching of black soil in Northeast China

**DOI:** 10.1038/s41598-020-71815-z

**Published:** 2020-09-10

**Authors:** Yujun Wang, Hongjun Gao, Zhonglei Xie, Lifeng Zhang, Xiulan Ma, Chang Peng

**Affiliations:** 1 College of Resources and Environment, Jilin Agricultural University, Changchun, 130118 Jilin China; 2 Institute of Agricultural Environment and Resources Research, Jilin Academy of Agriculture Sciences, Changchun, 130033 Jilin China; 3 College of Plant Science, Jilin University, Changchun, 130062 Jilin China; 4 College of Construction Engineering, Changchun Sci-Tech University, Changchun, 130600 Jilin China; 5 College of Tourism and Geographic Sciences, Jilin Normal University, Siping, 136099 Jilin China

**Keywords:** Environmental chemistry, Ecology, Environmental sciences

## Abstract

Considering the large amount and high frequency application of concentrated fertilizer nitrogen in the Black Soil Region of Northeast China, the current laboratory/field simulation study aimed to explore the pollution risk of added nitrogen in black soil to groundwater and identify effective measures to prevent and control soil nitrogen leaching with an undisturbed soil column. The results showed that the saturated nitrogen adsorption capacities increased by 1.7%, 7.7% and 18.5% in ploughing, impervious agent (starch grafted polyacrylic acid) addition, and corn straw returning treatments, respectively, relative to the control (no-till). When the collection volume of the leaching solution reached the experimental maximum (4,000 mL), the total amount of nitrogen leaching from the control soil column (i.e., the no-tillage treatment) accounted for more than 50% of the added nitrogen, indicating a great risk of nitrogen pollution in groundwater. Compared with the no-tillage treatment, the amount of nitrogen leaching from the ploughing treatment increased insignificantly, and the amount of nitrogen leaching in the following spring in the corn straw returning treatment increased by 11.2%. The amount of nitrogen leaching decreased by 12.5% in the soil sampled in autumn of the second year. The total amount of nitrogen leaching in the soil with impervious agents decreased by 40.1%. Therefore, the permeability-reducing agent could significantly reduce underground water pollution risk posed by nitrogen leaching.

## Introduction

The black soil located in the middle of the Northeast Songnen Plain, China, is one of the three famous black soil (Molisol) zones in the world. There is 8.15 million ha of cultivated black soil in Northeast China, which contributes to approximately 20% of the country's grain production^1^; therefore, black soil serves as an important commodity grain production base in China. Due to the relatively flat terrain of the black soil area, the proportion of non-point source pollutants (nitrogen and phosphorus) entering the water body with surface runoff is relatively low. However, the underground water pollution risk will increase through the vertical migration of nitrogen and phosphorus by leaching. Considering labour costs and planting habits, the farmers in this area mostly adopt a fertilization method called "one-time fertilization" (in which all fertilizers required for the whole growth cycle of crops are applied to the soil at one time when sowing in spring)^2^. This fertilization method will result in a low fertilizer utilization rate (generally no more than 35%) and a large amount of chemical fertilizer leaching to the groundwater^3^.

Nitrogen, one of the most important nutrient elements for plant growth, can not only be assimilated by crops but can also evaporate into gaseous forms via mineralization and denitrification and can be fixed with the organic matter of soil^4^. Other forms of nitrogen in soils not involved in the above processes may leach into the underground water^5–7^. In recent years, the leaching loss of soil nitrogen in cultivated land has become one of the major contributors to water pollution in many areas of China^8^, and the nitrogen pollution in water is closely related to land-use patterns and irregular events such as rainfall and soil disturbance; moreover, the leaching loss of soil nitrogen is difficult to control effectively^9–12^. Currently, the risk of nonpoint source pollution in the black soil region of Northeast China is increasing because the loss of nitrogen in black soil increased by 92% during 20 years^1, 13, 14^. Consequently, effective measures should be taken to control soil nitrogen leaching under the premise of maintaining the soil fertility of the black soil area in Northeast China^15^. An effective way to control nitrogen leaching in irrigated farming is to optimize irrigation management because controlling the irrigation amount and frequency will effectively limit the amount of water moving out of the active rooting area, thus reducing nitrogen leaching loss^16, 17^. However, in the black soil area, almost all farmland is rain-fed farming^18^, and optimized irrigation is not an option for controlling nitrogen leaching in the region.

In the black soil area of Northeast China, the degree of agricultural mechanization is increasing, and the no tillage method is increasing each year. The use of agricultural machinery and the no-tillage mode can lead to an increase in the degree of soil compaction; hence, some farmers are ploughing the land before sowing to soften the topsoil. After ploughing, the soil roughness, porosity and water storage capacity increase^18^, which can significantly inhibit surface runoff, and the sediment yield after ploughing decreases by more than 40% compared with no tillage^19^. The soil is loose, and the bulk density decreases after ploughing; however, whether it aggravates the vertical transport of nitrogen and increases the risk of groundwater pollution remain unclear. In the black soil area of Northeast China, surplus crop straw (mainly corn) in the field has become popular in recent years. The straw remaining in the field can lead to a decrease in soil bulk density and an increase in soil organic matter^20^. In addition, impervious agents added to soil have a certain inhibitory effect on nitrogen leaching^21^. Polyacrylic acid, an impervious agent, can keep water in soil because of its hydrophilic carboxyl group in its molecular structure; however, the cost of adding polyacrylic acid to the soil is relatively high, which limits its application in agriculture. Starch contains a large number of hydrophilic hydroxyl groups and has a strong water retention capacity. Grafting starch onto polyacrylic acid can greatly reduce the cost of using polyacrylic acid. Therefore, polyacrylic acid-grafted starch has been used as a soil water retaining agent^22^. The dissolving speed of starch-grafted polyacrylic acid as an impervious agent in the soil is faster than that of expansion and adhesion. Under the condition of sufficient soil moisture, the impervious agent in the surface layer dissolves and then penetrates into the soil, gradually expands and binds with other particles, blocking the space between the soil particles, thus playing a role of being impervious^23^. Consequently, the impervious agent can be directly applied to the 5–10 cm layer of soil to achieve an impervious effect in deep soil under high soil moisture content conditions^24^. Currently, the effect of straw remaining and impervious agent addition on nitrogen migration in the soil is lacking. Accordingly, the aims of this research were to (1) study the effects of ploughing, straw deep returning and adding an impervious agent on the physical and chemical properties of soil; (2) explore the adsorption capacity of soil to nitrogen; and (3) investigate the leaching characteristics of nitrogen in undisturbed soil columns.

## Results and discussion

### Effects of different agronomic practices on soil bulk density and organic matter content

**Figure 1:**
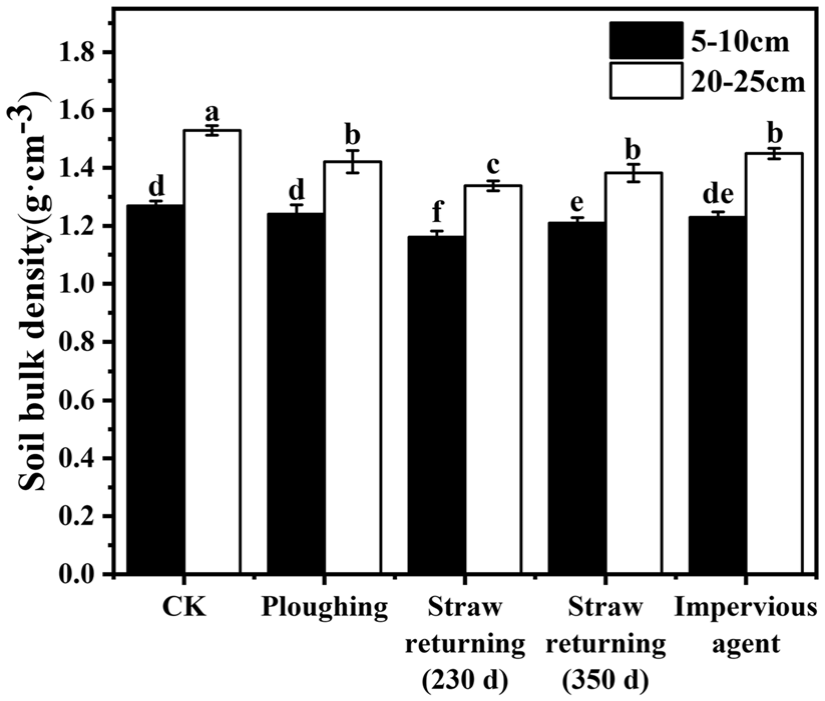
Effect of different agronomic practices on soil bulk density.

Soil bulk density can be used to evaluate the compactness and porosity of soil particles. Generally, the lower the soil bulk density is, the higher the porosity is, and the stronger the water permeability is^25^; consequently, the nitrogen leaching in the soil will be higher, and there will be a higher risk of groundwater pollution. The effect of different agronomic practices on soil bulk density is shown in Fig. 1. The bulk density of the 5–10 cm layer soil for CK (no-tillage control) was 1.27 g cm^−3^, which was higher than the 1.21 g cm^−3^ in the CK soil from the previous year. This result was mainly related to compaction from rainfall and mechanical operation. The subsoil bulk density (20–25 cm) of the no-till CK was 1.53 g cm^−3^, which was 16.6% higher than that of the topsoil (5–10 cm), which was mainly attributed to less disturbance in the subsoil than in the topsoil. Because of the disturbances of ploughing, straw returning and adding an impervious agent to the soil, the soil bulk density of all the above treatments decreased compared with that of the no-tillage CK. There was no significant difference between the ploughing and impervious agent addition treatments; however, straw returning significantly decreased the soil bulk density compared with that of no straw retuning (the simple tillage treatment). The soil bulk densities increased obviously with increasing time of crop return (230 days versus 350 days), which reflected differences in the extent of residue decomposition in soils, which decayed slowly in the first 230 days coupled with low temperature and had lower decomposition during the autumn, winter and spring compared with the quick decomposition period from 230 to 350 days (summer season) when the soil was wet and warm in the study area. The wetter and warmer soil conditions during the period from 230 to 350 days were associated with stronger microbial activity, which facilitated the rapid decomposition of corn straw, resulting in an increase in soil compactness and bulk density.

**Figure 2:**
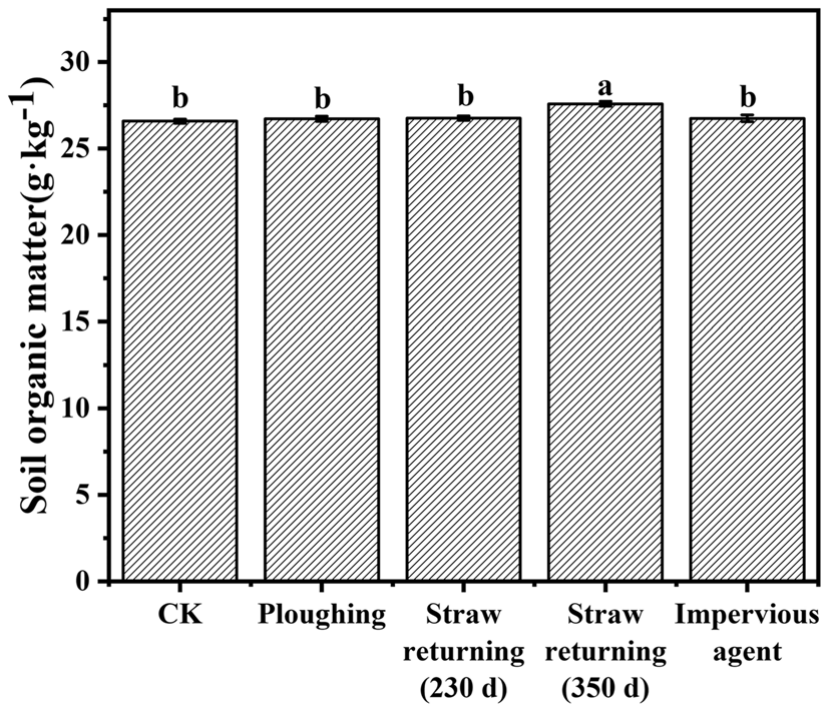
Effect of different agronomic practices on soil organic matter content.

Organic matter can improve soil structure, effectively hold water and crop nutrients, and effectively reduce leaching loss of nitrogen^25^. Figure 2 shows the effects of different agronomic practices on the contents of soil organic matter. Non-decomposed corn straw was visible in the soils after 230 days of corn straw returning and should be removed before organic matter determination from the soil samples. However, non-decomposed corn straw was invisible from the soil after 350 days of corn straw returning. It is believed that a portion of corn straw in the soil was converted into the soil organic matter pool, resulting in soil organic matter that was 3.8% higher after 350 days of straw returning than that in the control. Although much straw did not convert into soil organic matter after 230 days of straw returning, this kind of organic material probably had an impact on the migration of soluble substances in the soil^26^.

### Adsorption characteristics of nitrogen in cultivated soil treated with different agronomic practices

**Figure 3:**
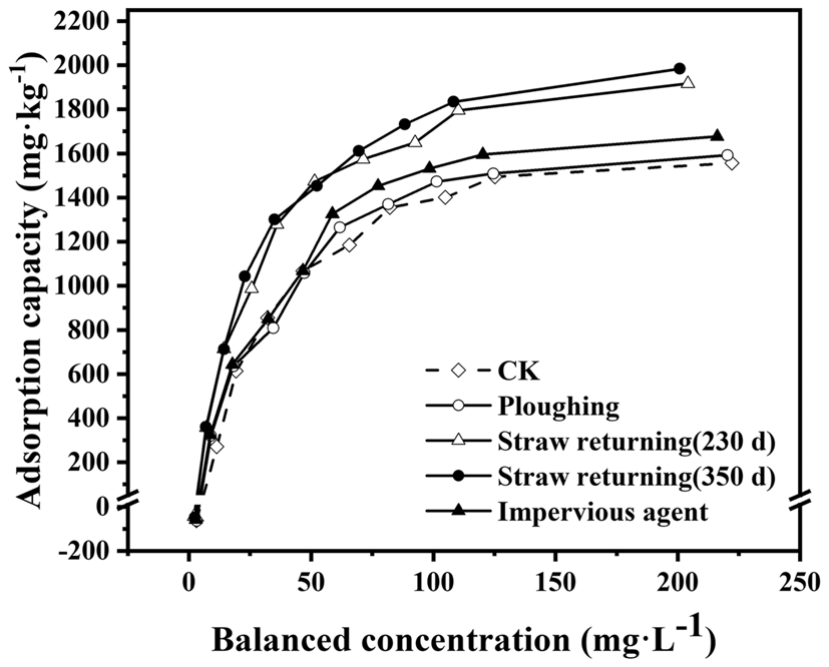
Adsorption isotherm curve of nitrogen in soils treated with different agronomic practices.

Figure 3 shows the adsorption isotherm curves of nitrogen in black soil with different agronomic practices under a constant temperature of 25 °C. The initial adsorption capacity of each treatment without exogenous nitrogen addition was negative, which was mainly caused by nitrogen in the soil dissolved into the solution^22^. When the concentration of exogenous nitrogen increased, the initial dissolved nitrogen in the soil could be ignored. In the low range of nitrogen concentrations (< 125 mg L^−1^), the adsorbed nitrogen in the soil increased sharply with increasing nitrogen concentration in the solution. When the equilibrium concentration of nitrogen reached a certain upper limit (> 125 mg L^−1^), the adsorbed amount of nitrogen in the soil did not increase with an increase in the nitrogen concentration of the solution, which indicated that the adsorption capacity of nitrogen in black soil reached equilibrium.

**Figure 4:**
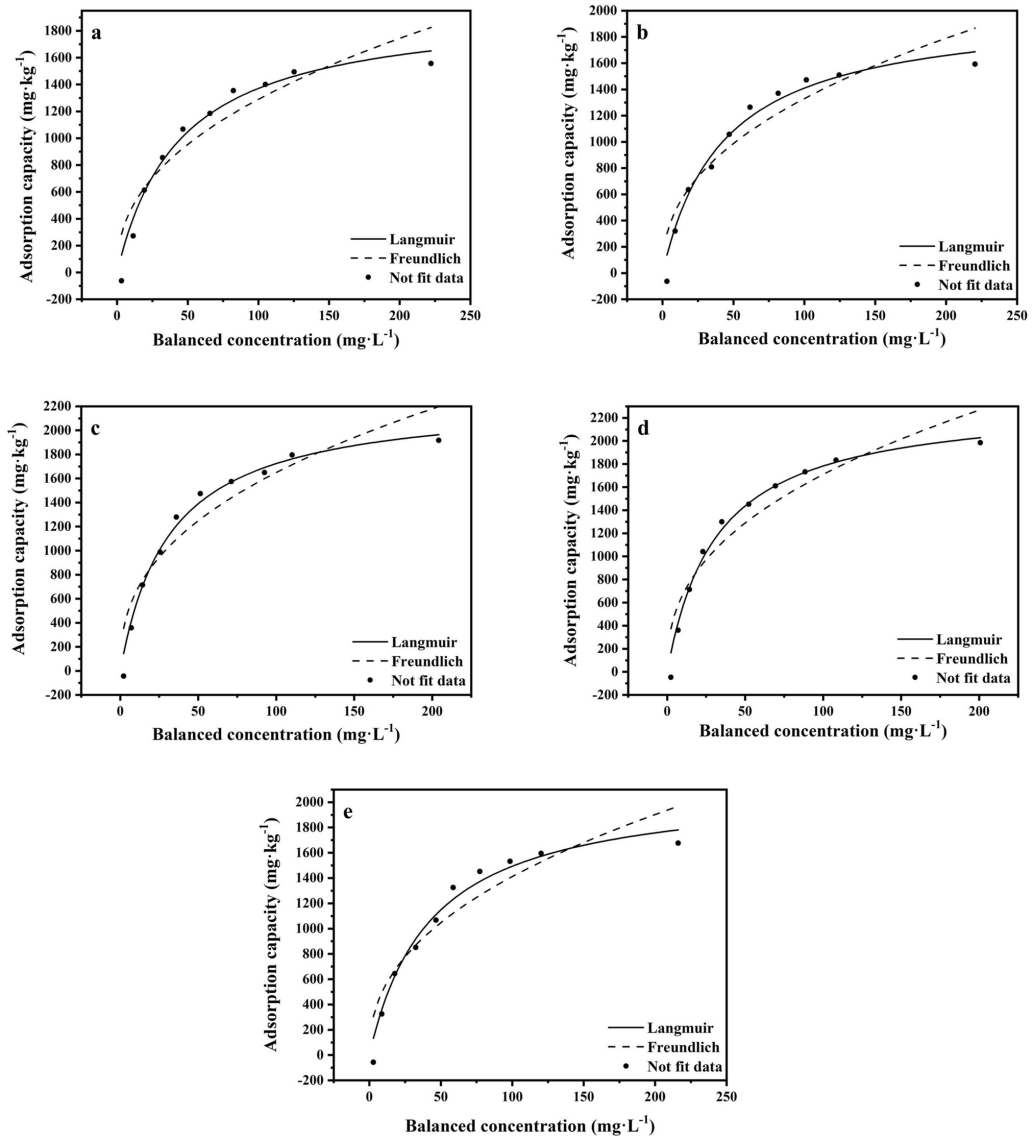
Fitting curve of nitrogen adsorption isotherm in different soils. (**a**) CK, (**b**) ploughing, (**c**) straw returning (230 days), (**d**) Straw returning (350 days), (**e**) impervious agent.

The adsorption characteristics of nitrogen in the soils were fitted with different models under different agronomic practices. Fitting could be done using the linear Henry equation in the lower nitrogen balance concentration and with the Freundlich or Langmuir equations in the higher nitrogen balance concentration^27^. Figure 4 is the curve of the Freundlich and Langmuir equations of nitrogen adsorption in the soils. The Langmuir equation deviates less from reality; further, the fitting results in Table 1 are consistent with those in Fig. 4.

**Table 1:** Fitting results of adsorption thermodynamics equation of nitrogen in tested soils.

Treatment	Langmuir		
	*k*_*L*_	*Q*_*e*_	*r*
CK	0.02247	1981.3	0.9848**
Ploughing	0.02328	2015.1	0.9857**
Straw returning (230 days)	0.03167	2,267.9	0.9920**
Straw returning (350 days)	0.03155	2,347.6	0.9918**
Impervious agent	0.02330	2,134.6	0.9868**

** indicates an extremely significant correlation (*p* ≤ 0.001)

According to the Langmuir equation, the saturated adsorption capacity (Q_*e*_) of nitrogen in the soils increased by 1.7%, 14.5%, 18.5% and 7.7% for the soils treated with ploughing, straw returning for 230 days and 350 days, and addition of an impervious agent, respectively, compared with that of the no-tillage treatment. The nitrogen adsorption capacity in ploughed soil increased insignificantly, which may be related to the fact that the plough mainly changed the soil physical properties (e.g., bulk density and permeability) but had no significant effect on the chemical composition and soil colloidal properties. Nevertheless, there was a slight increase in the adsorption capacity of nitrogen in the soil treated with impermeable agents. The result may be because certain functional groups, such as hydroxyl groups, carboxyl groups and starch and acrylic acid originating from the impermeable agent, enable the formation of a network structure with nitrogen and contribute to the adsorption of nitrogen^28^. The adsorption capacity of nitrogen increased by 14.5% in the corn straw treatment (230 days), which may be because the straw in the soil just started to decay and had not been converted into soil organic matter^28^. Moreover, the straw that had decayed partially had a large specific surface area, which could contribute to the adsorption of nitrogen in soil. The adsorption capacity of nitrogen increased by 18.5% 350 days after straw was returned to the field, indicating that the newly formed organic substance from straw had a great adsorption capacity for nitrogen. Furthermore, dissolved soil organic matter can enhance the adsorption of nitrogen in soils through ion exchange, adsorption and chelation^29^.

The adsorption of nitrogen in soils is affected directly and indirectly by soil organic matter^30^. The direct effect is that soil organic matter forms a relatively stable particle complex with nitrogen through physical and chemical adsorption or forms a more stable chelate with dissolved nitrogen through complexation^31^, and the indirect effect is by adjusting the physical and chemical properties of soil, such as pH and Eh, or by making nitrogen reach the soil surface or enter the soil interior easily, which results in an increase in the adsorption of nitrogen in soils. Therefore, the application of organic substances can increase the nitrogen holding capacity of soil and mitigate the risk of groundwater pollution by reducing the leaching loss of nitrogen in soils.

### Leaching characteristics of nitrogen in soil under different agronomic practices

**Figure 5:**
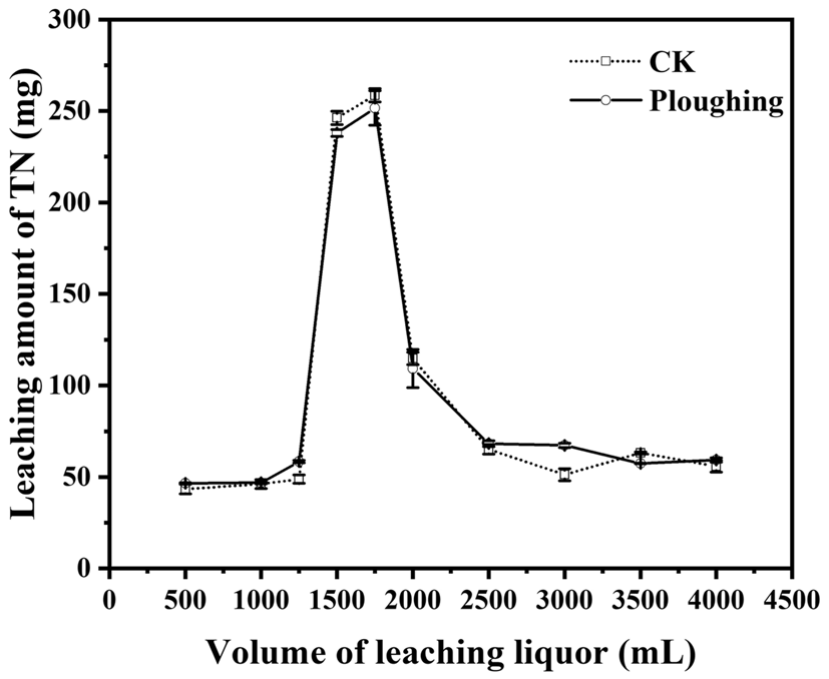
Effect of ploughing treatment on nitrogen leaching in the soil.

**Figure 6:**
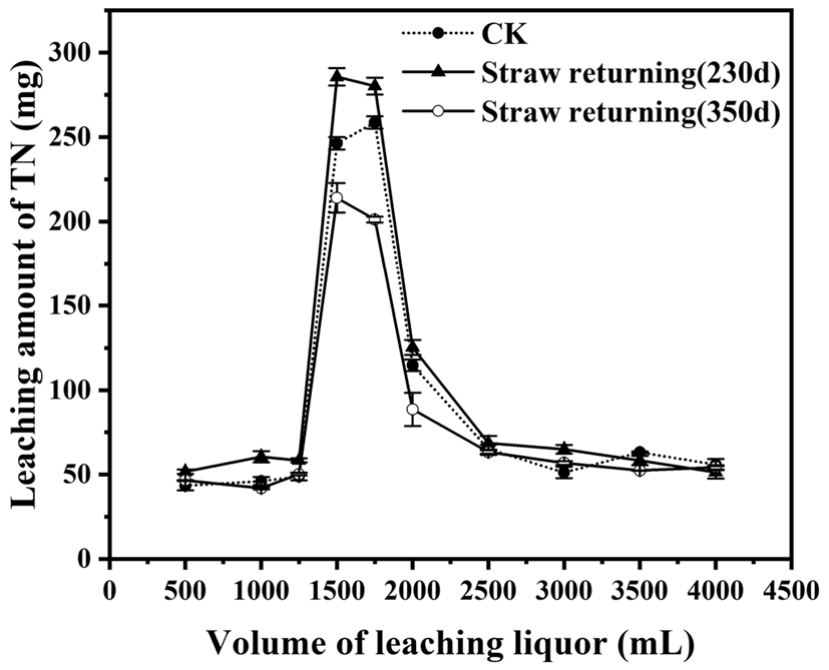
Effect of straw returning on soil nitrogen leaching.

**Figure 7:**
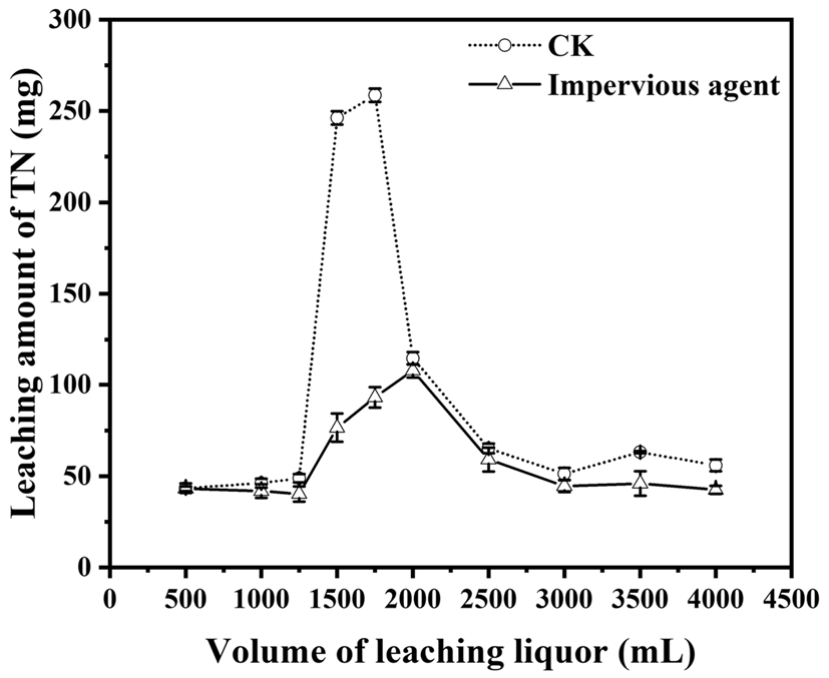
Influence of adding the impervious agent on nitrogen leaching.

The same amount of leaching water (4,000 mL) was collected from each leaching column with a total of 10 sampling segments (subsamples) as described in the Materials and Methods section. The majority of leached nitrogen was recovered in the 4th and 5th samples after 1,250 mL of leaching water passed through the column, with the same trend for all treatments (Figs. 5, 6, 7). Another similar feature for all treatments was that only a small amount of nitrogen (< 150 mg, approximately 15% of the total nitrogen addition) was recovered in the first three leachate samples (1,250 mL), and it was certain that the nitrogen was not from added urea but from residual soil nitrogen.

#### CK

A total amount of 993.5 mg nitrogen was collected from this treatment, which was more than 50% of the added nitrogen. More than 50% of the leached nitrogen occurred in the 4^th^ sampling segment (1,250–1,750 mL). Although nitrogen in soil is highly soluble, the amount of nitrogen leaching in the soils in this experiment was not particularly high, and nearly 50% of nitrogen remained in the soil. The results were similar to Chen's study on nitrogen transport in black soil in this area^31^, and this similarity may reflect the low amount of residual nitrogen in the soil column after pre-leaching and the strong adsorption capacity of soil colloids for nitrogen^32^.

#### Ploughing treatment without corn straw return

Without corn straw return, there was no difference in nitrogen leaching between the ploughing and no-tillage treatments (Fig. 5) (t-test, P > 0.05), indicating that ploughing (top 10 cm) had no significant impact on the performance of nitrogen leaching and would not increase the risk of nitrogen pollution to groundwater. Although the soil bulk density decreased significantly and the soil structure was loose after ploughing, which was conducive to nitrogen leaching^33^, proper ploughing can destroy the large pore space of the arable soil, inhibit the generation of soil preferential flow, block the migration of nitrogen to groundwater, and reduce nitrogen leaching. These confounding effects led to insignificant changes in the characteristics of nitrogen leaching in ploughed soils compared with no-tillage soils.

#### Ploughing treatment with straw returning

Figure 6 shows the leaching results from two sets of soil columns. One set of soil was collected after 230 days of corn straw addition, and another was collected after 350 days of straw addition. There was more nitrogen leaching (1,105.2 mg N) in the former and less nitrogen leaching (869.6 mg N) in the latter compared with that in the control (993 mg N). This difference was probably due to the difference in corn straw status in the soil. For the soil column collected after 230 days of straw return, the straw was decomposed partially under the conditions of the low temperature and soil moisture in early spring^3^. Non-decomposed straw also resulted in a lower soil bulk density, higher soil porosity, higher permeability and more nitrogen leaching out of the column compared with that of the soil column collected after 350 days of corn straw returning, in which the corn straw had experienced more complete decomposition during the wet and warm summer. More humified organic matter from decomposed straw had a high cation exchange capacity relative to un-humified organic substances. It was confirmed that decomposed straw was more beneficial to prevent the loss of nitrogen in black soil. If the straw was returned to the field after the autumn harvest, excessive fertilization should be prohibited in the sowing period to reduce the loss of nitrogen due to the incomplete decomposition of straw and the strong leaching of soil nitrogen^34^. Of course, fertilization methods also had a great influence on nitrogen leaching solution; for example, the “one-time fertilization” approach, which has been widely used in the studied area, was a very environmentally unfriendly way of fertilization and should be prohibited^35^.

#### Treatment of adding impervious agents

The effect of adding impervious agents on the rate of drenching solution is shown in Table 2*.* The results of the pre-leaching experiment showed that there was no significant difference in the rate of water infiltration between the treatments with and without impervious agents, which may be related to the lower soil moisture content, the less obvious expansion of the impervious agent molecules and the lower blocking effect on the soil voids. When the leaching experiment started and the collection volume of the leaching solution reached 4,000 mL without limiting the outflow, the average flow rate of the leaching solution was 3.8 mL min^−1^ for the impervious agent addition treatment, which was significantly lower than that of the control treatment (6.5 mL min^−1^). This result was mainly related to the strong blocking effect of the expansion of impervious agents in the soil void after a long period of water immersion.

**Table 2:** The effect of adding impervious agent on the rate of drenching solution.

Treatment	Pre leaching stage (mL min^−1^)	Leaching stage after nitrogen application (mL min^−1^)
CK	6.6 ± 0.2 a	6.5 ± 0.2 a
Impervious agent	6.5 ± 0.1 a	3.8 ± 0.1 b

Figure 7 shows that the peak of nitrogen leaching was delayed with impervious agent treatment relative to the control. The peak and total leaching amounts of nitrogen were 107.8 mg and 594.7 mg, respectively, which were significantly lower than that of the control (250 mg nitrogen for peak and 993 mg nitrogen for total). Therefore, the effect of impervious agents on preventing soil nitrogen leaching was obvious, and starch-grafted polyacrylic acid, as an impervious agent, can effectively inhibit the infiltration of soil water and the leaching loss of soil nitrogen.

## Conclusions

Compared with the control (no tillage), tillage had no significant effect on the soil organic matter content, nitrogen adsorption capacity or leaching amount in black soil. The soil organic matter content and nitrogen adsorption capacity of the soil were significantly increased for the straw returning treatment. Due to the influence of straw decay degree, the leaching amount of soil nitrogen increased by 11.2% when straw was returned to the field for 230 days and decreased by 12.5% when straw was returned to the field for 350 days. The addition of an impervious agent (starch grafted polyacrylic acid) did not increase the content of soil organic matter but could enhance the adsorption capacity of nitrogen and significantly reduce the leaching loss of nitrogen in black soil.

## Materials and methods

### General situation of the research area

**Figure 8:**
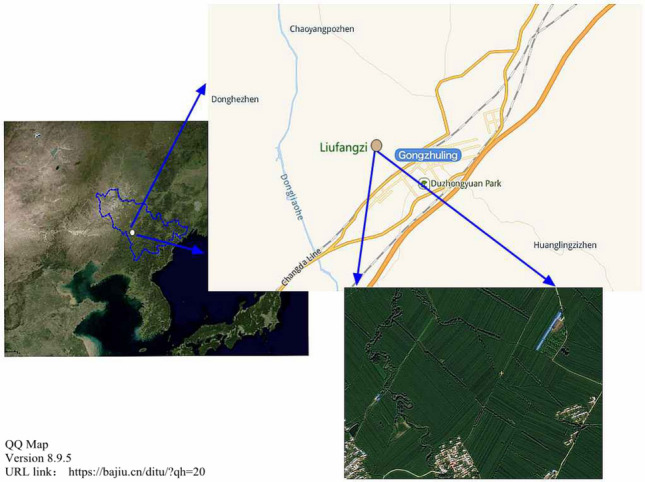
Location of study area (Liufangzi Village, Gongzhuling City, Jilin Province).

The research area was conducted at Liufangzi village, Gongzhuling city, Jilin Province (N43°34′10″, E124°52′55″), as shown in Fig. 8. The area has a continental monsoon climate in the humid area of the middle temperate zone, with an average annual precipitation of 594.8 mm, which is mainly concentrated in June and August. The average annual temperature is 5.6 °C, and the daily average temperature drops to 0 °C in November of each year, with a freezing period of up to five months. Corn is one of the main commodity crops in the area, with a sowing date in early May and a harvest date in early October.

The soil of the site is a silty loam black soil, which had been planted with monoculture corn with no tillage for 5 years. On October 5, 2018 (after the autumn harvest), a flat field was selected to set up the experiment. Soil samples were collected using the zigzag sampling method, and selective physical and chemical properties of soil were determined, including pH (5.48), organic matter (26.4 g kg^−1^), clay (29.12%), and soil bulk density (1.21 g cm^−3^ in 5–10 cm and 1.53 g cm^−3^ in 20–25 cm).

### Reagents and instruments

#### Reagents

The main raw material of the added impervious agent was corn starch and acrylic compound, which was entrusted to Jilin Yida Chemical Co., Ltd. The added urea was an analytical reagent, and the reagents used for analysis included H_2_SO_4_, H_3_PO_4_, NaOH, NH_4_OH, NH_4_Cl, K_2_S_2_O_8_, Na_2_B_4_O_7_, KNO_3_, KNO_2_, K_2_Cr_2_O_7_, FeSO_4_, sulfonamide, and naphthalene ethylenediamine hydrochloride; these were all analytical reagents provided by Beijing Chemical Plant.

*Instruments* laboratory-built soil leaching column; continuous flow injection analyser (SKALAR SA++, Netherlands).

### Test plot setup and agronomic practices

The experimental plots were maintained in the field consisting of (1) CK (no-tillage control treatment, with corn straw removed and soil left under no-till management); (2) ploughing treatment (corn straw was removed and then mouldboard ploughed to a 30 cm depth); (3) straw returning treatment (corn straw (25.32% moist) was incorporated into the soil on October 5, 2018 (after autumn harvest), with an application amount of 1.25 kg m^−2^. Briefly, corn straw was chopped into small pieces (0.5 cm length), evenly placed on the soil surface, and then incorporated into the soil with ploughing (the depth of 30 cm)); and (4) impervious agent addition treatment (the impervious agent mentioned previously evenly laid on the soil surface at the amount of 15 g m^−2^ and then incorporated into the 0–30 cm soil by mouldboard ploughing). The abovementioned field operations were conducted after corn harvest in the fall of 2018 with a testing area of 10 m × 50 m for each plot and three replicates for each treatment. In the following spring (2019), grain corn was planted in all treatment plots with a planting density of 65,000 plants ha^−1^. All plots were managed in the same way with a one-time fertilization application of 200–90-90 kg (N-P-K) ha^−1^ and 2,4-d spray as weed control.

**Figure 9:**
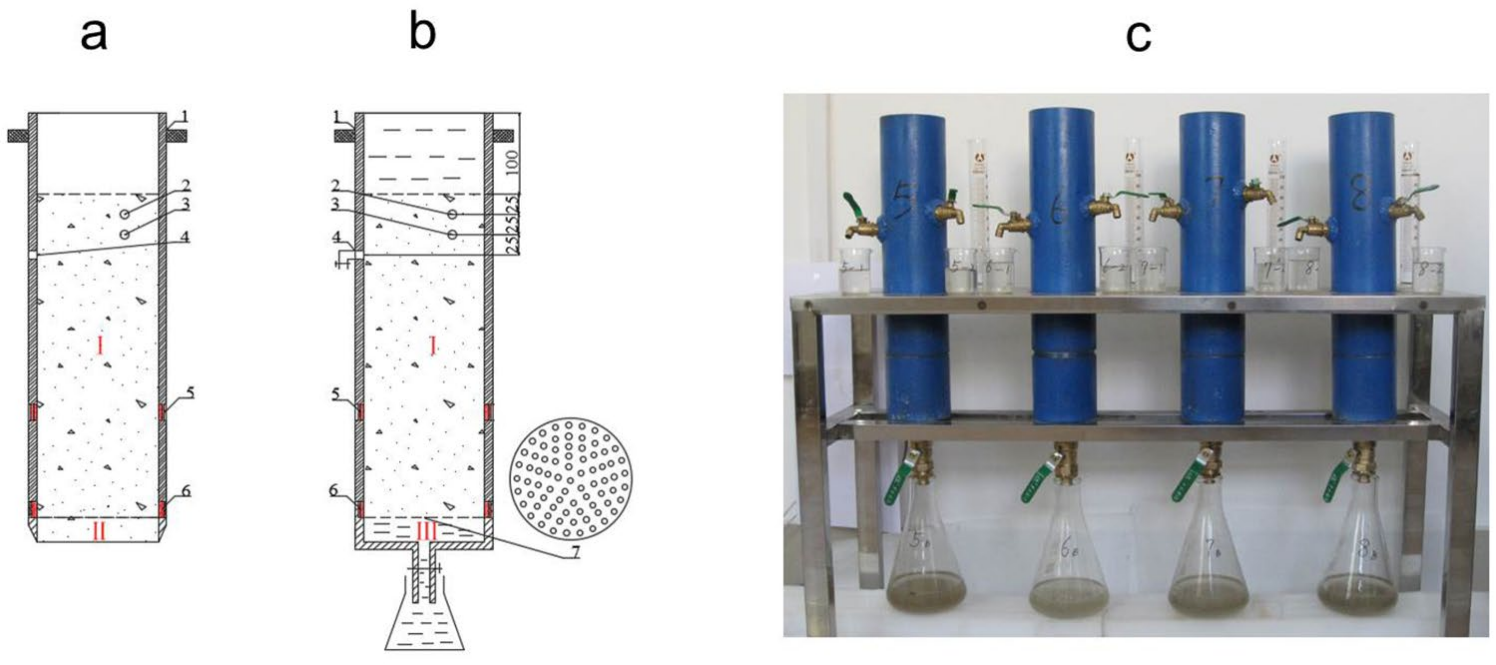
Schematic diagram of simulated leaching device of undisturbed soil column. (**a**) Soil extraction; (**b**) leaching; (**c**) physical map of leaching in undisturbed soil column. 1: Handle; 2.3.4: guide port; 5.6: screw port; 7: punching plate. I main body of leaching column; II soil cutter; III leaching solution collector.

For all the above treatments (including the control treatment), undisturbed soils (0–30 cm layer) were collected with an undisturbed soil column (refer to Fig. 9) for the leaching experiment on September 25, 2019 (before autumn harvest, after 350 days of straw returning to the field); soil samples of 0–15 cm were collected for determination of soil organic matter and adsorption experiment of nitrogen in the soil; and soil samples of 5–10 cm and 20–25 cm layers were collected for determination of soil bulk density. In addition, for the straw returning treatment, one sampling was added on May 25, 2019 (one month after sowing, 230 days after straw returning), for the determination of soil organic matter content and soil bulk density, nitrogen adsorption and leaching experiment in soil.

The soil samples used for soil organic matter determination and nitrogen absorption testing were air dried, sieved through a 2-mm sieve and visible plant debris and stones were removed, and then stored.

### Experiment of nitrogen adsorption in soil

Ten parts of the soil samples (air-dried, < 2 mm and 1.00 g per part) were weighed into a triangular flask (numbered 1 to 10), and then 20.00 mL of urea solution with different nitrogen concentrations of 0, 25, 50, 75, 100, 125, 150, 175, 200, and 300 mg L^−1^ was added to each flask numbered 1 to 10, respectively. The flasks were placed in an incubator with oscillator shaking for 4 h at a constant temperature of 25 ± 1 °C, and then the supernatant was collected to determine the total nitrogen in the solution. This test was performed with 3 replicates for each treatment.

### Nitrogen leaching test with undisturbed soil column

#### The structure of the leaching column

The leaching column was made of stainless with an inner diameter of 10 cm and three parts: part I (the main body of the leaching column), part II (the soil cutter, used for soil core collection) and part III (the collector of leaching solution, used for leachate collection) (see Fig. 9). The height of the leaching column was 40 cm, with the top 10 cm empty for holding leaching solution and the bottom 30 cm was filled with undisturbed soil.

#### Collection of undisturbed soil columns

The bottom of part I was connected with part II, and the undisturbed soil column was collected by pressing the handles vertically. When the soil surface of the soil column was 10 cm away from the upper end of the column, the sampling was finished. To reduce the soil disturbance and resistance during the sampling process, the soil surrounding the targeted soil column was first removed carefully with a spade (manually) and the diameter of the soil column was left slightly larger than the inner diameter of the leaching column, watered evenly and slowly; then, the leaching column was carefully placed on top of the soil column and gently pressed down to the required depth.

#### Leaching method of nitrogen in undisturbed soil column

1. *Pre-leaching and exogenous nitrogen addition* The soil in the collected undisturbed soil column of part II was scraped off, part II was removed, and part III was connected to part I (see Fig. 9b). The flow control valve at the three diversion ports was connected at the upper end of component I to simulate the surface runoff under the field conditions, and the leaching speed was controlled through the flow valve at the lower end (part III). Before the nitrogen leaching experiment, the soil column was pre-leached with 3,000 mL deionized water to remove soil residual nitrogen. The eluent was discarded, and the soil column was placed for 48 h before the start of the leaching experiment.
  The top 1 cm of undisturbed soil was collected as the covering soil, and then 2 cm of additional undisturbed soil was taken and mixed thoroughly with 4.0 g of urea. The soil-urea mixture was installed back into the leaching column, covered again with the covering soil, and then placed for 4 h before the leaching test.
2. *Leachate collection* The leaching test was conducted by adding deionized water to the soil leaching column. The leaching process was carried out continuously until the projected volume of leachate was achieved. The total volume of projected leachate collection was 4,000 mL, roughly equivalent to the average annual precipitation (510 mm) in the study area. In total, 10 leachate samples were collected from each column, and the sample volumes varied from 250 mL each for the 3rd to 6th samples and 500 mL each for the rest of the samples. To eliminate the influence of temperature on the leaching process, the leaching test was carried out in a constant temperature chamber at 25 ± 1 °C.

### Analysis method

Soil bulk density was determined with the ring knife method. Soil organic matter and total nitrogen contents were analysed using the potassium dichromate volumetric method and N-(1-naphthyl) ethylene diamine dihydrochloride spectrophotometry, respectively^36^.

### Data processing methods

#### Adsorption capacity

The amounts of nitrogen adsorbed by the soils were determined using the following equation (Eq. 1):

1 $$Q = (c_{0} - c_{e} )V/m$$

where *Q* (mg L^−1^) is the adsorption capacity, *c*_*0*_ (mg L^−1^) denotes the initial solution concentration of the adsorption test, *c*_*e*_ (mg L^−1^) represents the concentrations of the adsorption equilibrium solution, and “*m*” refers to the soil quantity (g)^27^.

Two isothermal models were used to calculate the nitrogen adsorption capacity (Eqs. 2 and 3):

2 $${\text{Langmuir}}\;{\text{equation}}:\;Q = k_{L} Q_{e} c_{e} /(1 + k_{L} c_{e} )$$

3 $${\text{Freundlich}}\;{\text{equation}}:\;Q = k_{F} c_{e}^{1/n}$$

where *Q* (mg L^−1^) is the adsorption capacity, *c*_*e*_ (mg L^−1^) represents the concentration of the adsorption equilibrium solution, *Q*_*e*_ (mg L^−1^) refers to the saturated adsorption, and *k*_*L*_, *k*_*F*_, and *n* are constants related to adsorption capacity^27^.

Microsoft Excel 2010 was used for data processing and statistical analysis, and Origin 8.5 software was used to fit the data. T-test was used to test the difference between the two groups.
